# Synthesis and degradation kinetics of TiO_2_/GO composites with highly efficient activity for adsorption and photocatalytic degradation of MB

**DOI:** 10.1038/s41598-019-54320-w

**Published:** 2019-12-10

**Authors:** Ruifen Wang, Kaixuan Shi, Dong Huang, Jing Zhang, Shengli An

**Affiliations:** Inner Mongolia Key Laboratory of Advanced Ceramic Materials and Devices, School of Materials and Metallurgy, Inner Mongolia University of Science and Technology, Baotou, 014010 PR China

**Keywords:** Materials science, Materials for energy and catalysis, Photocatalysis

## Abstract

Poriferous TiO_2_/GO (denoted as TGO-x%) photocatalysts with ultrathin grapheme oxide (GO) layer were prepared by a hydrothermal method, the adsorption and photocatalytic degradation and its kinetics about Methylene blue(MB) were studied systematically. All the TGO-x% showed improved adsorption and photodegradation performance. TGO-25% had excellent adsorptivity while TGO-20% exhibit the highest visible light photocatalytic degradation activity. The adsorption capacity for TGO-25% was 20.25 mg/g_catalyst_ along with the k_1_ was about 0.03393 min·g_catalyst_/mg, this enhancement was mainly owing to the strong adsorption capacity of GO and the stacking structure of sheets and nanoparticles. GO sheets prevented the agglomeration of TiO_2_ particles and TiO_2_ nanoparticles also prevented the agglomeration of GO sheets, which could provides greater surface area. Besides, the remarkably superior photodegradation activity of TiO_2_/GO composites is mainly attribute to the strong absorption of visible light and the effective charge separation revealed by the photoluminescence, the total removal rate of MB is 97.5% after 35 min adsorption and 140 min degradation, which is 3.5 times higher than that of TiO_2_.

## Introduction

For its high effectiveness and no secondary pollution, the photocatalytic oxidation technology has been regarded as the most appealing method in wastewater treatment. Among various photocatalysts, TiO_2_ is one of the most promising photocatalysts due to its high oxidative ability, cost-effectiveness, physical and chemical stability^1,2^. However, the inherent properties of TiO2 such as large band gap (~3.2 eV), low quantum efficiency and poor pollutant removal capacity lead to its performance in photocatalytic reactions is still very low, and greatly limits its applications in waste water purification^3,4^. Therefore, it’s urgently needed to develop a new type of photocatalyst with high pollutant removal capacity. Various strategies have been employed to solve these problems, such as doping with metal or nonmetal elements^5,6^, coupling with semiconductors^7,8^ or co-catalysts^9^, and recombination with carbon-based nanomaterials such as carbon nanotubes^10^, graphite oxide (GO)^11,12^, and graphene^13,14^.

Because of intrinsic stoichiometry, graphene oxide nanosheets are anionic two-dimensional materials with large surface areas, good mechanical strength and optical properties^15^. Moreover, GO is a hydrophilic substance with good electronic conductivity and electronic mobility. The unique structure makes graphene oxide an excellent co-catalyst or catalyst support, especially photocatalysts^16,17^ for removal and photocatalytic degradation of pollutants. It is presumable that the use of GO as an effective electron mediator can be readily extended to design and synthesize novel semiconductor-based composite photocatalytic systems for applications in energy and environmental science^18,19^.

TiO_2_-graphene with 3D network structure and TiO_2_-graphene hydrogel electrodes have been synthesized via a one-pot method^20^ and the photoelectrocatalytic ability over these electrodes was studied in a dynamic system. The electrodes exhibit strong adsorption-enrichment for pollutants for its large specific surface area and interconnected abundantly porous channels. Tong Z and the co-workers^21^ assembled a porous g-C_3_N_4_/graphene oxide aerogel via a hydrothermal method, and the composite photocatalyst showed remarkably improved activity for methyl orange degradation. However, 3D network, the aerogel, or other special structure photocatalysts are complex or difficult to synthesize, and it remains a challenge to prepare graphene-based photocatalysts by simple method and meet the deep mineralization ability for organic pollutants. Here, we report the fabrication of GO/TiO_2_ composites using a one‐step hydrothermal method, the percentage of GO in the composites were optimized based on the composite’s performance in the photocatalytic degradation of methylene blue (MB), and the adsorption/photocatalytic activity and kinetics have been discussed in detail. The GO/TiO_2_ composites possess large specific surface area and effective charge carrier separation ability, which greatly improved the adsorption capacity for organic pollutants. This study provides an effective method for the construction of TiO_2_ based composite with efficient photocatalytic mineralization ability and facilitates their potential application for water purification.

## Experimental

### Synthesis of TiO_2_ and GO

All chemical reagents for synthesis were analytically pure without further purification. TiO_2_ was synthesized by the sol-gel method. Briefly, the homogeneous mixture of 17 mL tetrabutyl titanate and 50 mL alcohol was added dropwise into the mixture of 30 mL alcohol, 10 mL glacial acetic acid and 10 mL distilled water, the mixture was stirred continually for another 1 h. After aging for 24 h in 343 K water bath, the obtained sol was dried in a thermostatic drying oven at 363 K for 12 h and then calcined at 773 K for 2 h to get the TiO_2_ powder.

GO was prepared from natural graphite powder using a modified Hummers method. Typically, a 3-neck flask equipped with Teflon coated magnetic stir bar and reflux condenser was placed with concentrated sulfuric acid (69 mL) and was cooled in an ice-water bath. Graphite powder (3.0 g) was dropped slowly into the flask, then sodium nitrate (1.5 g) and potassium permanganate (9 g) were gradually added to the mixture in sequence. After stir continuously in the ice-water bath for 2 h, the flask containing the mixture was transferred to a 308 K water bath and stirring was continued for 2 h. Under gently stirring, water (120 mL) was added then the mixture was heated to 368 K and reacted for 15 min. Water (300 mL) and H_2_O_2_ aqueous solution (5 mL 30%) were added to dilute the mixture. 20 min later, the suspension was centrifuged at 8000 RPM and washed with aqueous hydrochloric acid solution (10 wt%) until no sulfate ion was detected. The obtained GO was dried in a vacuum oven at 333 K for 12 hours.

### Synthesis of TiO_2_/GO composites

The TiO_2_/GO composites were synthesized using a hydrothermal method. Specifically, a certain amount of GO was homogeneous dispersed into 50 mL water under sonication for 30 min, then the solution was added into 70 mL TiO_2_ aqueous suspension (7.14 mg/mL) by dripping slowly with continuous stirring, stired continuously for 1 h. Next, the mixture was transferred into a 200 mL Teflon-lined stainless-steel autoclave and heated at 403 K for 12 h. Naturally cooled to room temperature, the black precipitates were collected by centrifugation, washed alternately with deionized water and ethanol several times, then dried in a vacuum oven at 333 K for 8 h. A series of samples were prepared by changing the weight of GO (m = 0, 26.31 mg, 55.55 mg, 88.23 mg, 125 mg and 166.67 mg), which were denoted as TiO_2_, TGO-5%, TGO-10%, TGO-15%, TGO-20% and TGO-25%, respectively. For comparison, TiO_2_ powders were also reprocessed using the hydrothermal method.

### Material characterization

Crystal structure of the samples was determined by an X-ray diffractometer (XRD, D8 Advanced, Bruker, Germany) with Cu Kα radiation (λ = 0.15418 nm). Morphology of the samples was characterized by a field emission scanning electron microscope (SEM, S-3400N, Hitachi, Japan). The structural features and high resolution transmission electron microscope (HR-TEM) photograph of samples were investigated on a (JEOL, JEM-2100) field emission transmission electron microscopy, which was equipped with an energy dispersive X-ray spectrometer (EDS) and mapping for elemental analysis, with 200 kV accelerating voltage. The Brunauer-Emmett-Teller (BET) surface area of samples were measured at 77 K using a surface area analyzer (Microtrac BEL, BELSORP-mini II). Fourier transform infrared spectra (FT-IR) was recorded using an FT-IR spectrometer (Tensor II, Bruker, Germany). The Raman spectra was recorded at room temperature using a Raman spectrometer (XploRA PLUS, Jobin Yvon, France) with an excitation of 532 nm^22^ laser light. X-ray photoelectron spectroscopy (XPS) measurements were obtained with an Multifunctional imaging electron spectrometer Thermo (ESCALAB 250XI) system. Ultraviolet-visible (UV-vis) absorption spectra at room temperature between 200–800 nm of the samples were obtained on a spectrophotometer (U-3900, Hitachi, Japan.) using Al_2_O_3_ as the reference. The Photoluminescence (PL) spectra was recorded on a Fluorescence Spectrophotometer (F-4600, Hitachi, Japan).

## Results and Discussion

### Morphology and phase characterization

**Figure 1:**
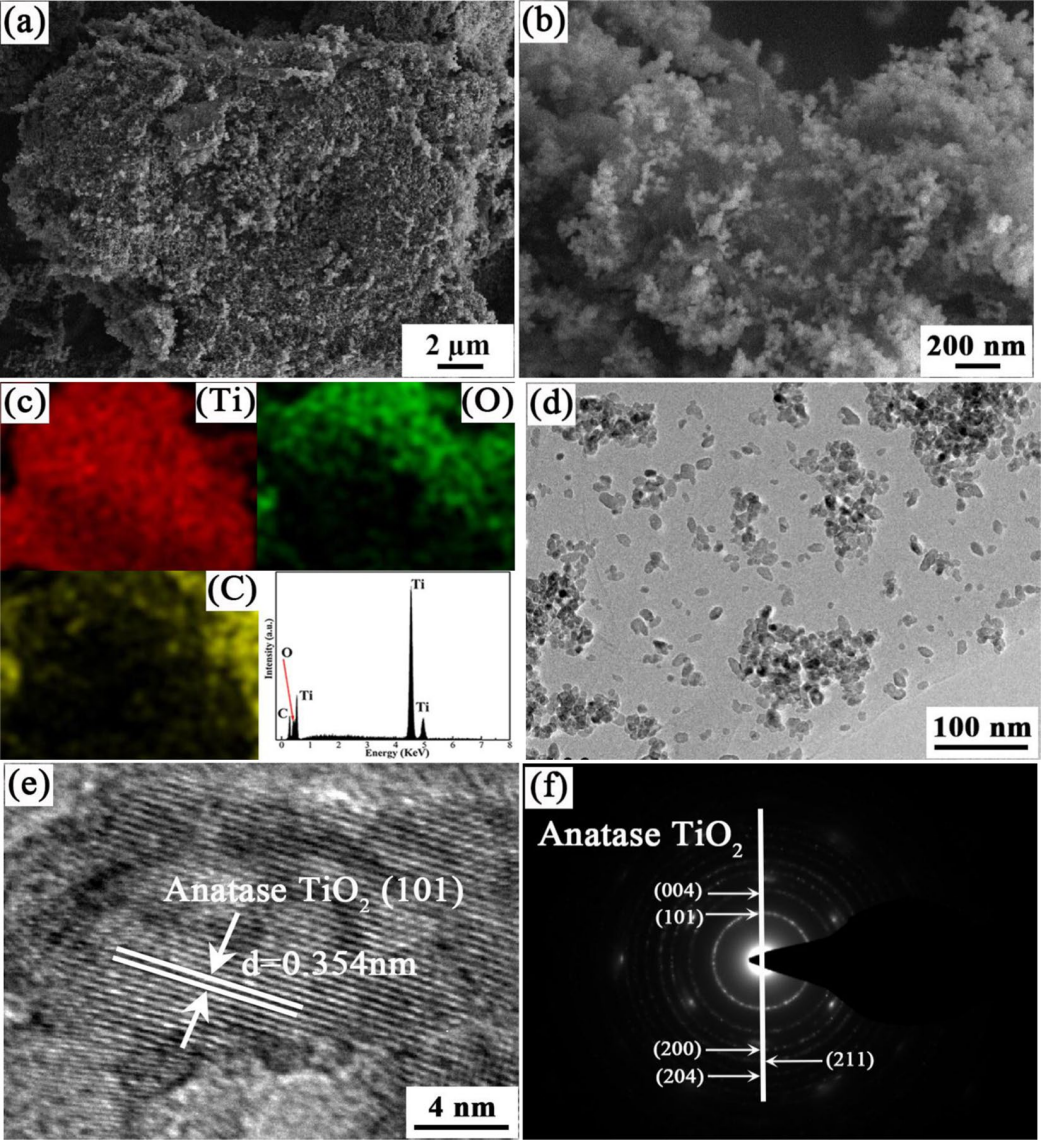
SEM (**a**,**b**), EDS mapping (**c**), TEM (**d**), HRTEM (**e**) and SAED (**f**) images of TGO-20%.

The morphology and microstructure of sample was observed by the FE-SEM and TEM. As shown in Fig. 1(a,b), a large number of small and uniform TiO_2_ particles located on the graphene oxide sheets and distributed evenly. Some ultrathin graphene nanosheets could be seen clearly. Importantly, these TiO_2_ particles showed a considerably uniform dispersion on the graphene sheets surface, the EDS mapping images as seen in Fig. 1(c) of TGO-20% showed Ti and C atoms are well dispersed. TEM images as seen in Fig. 1(d) displayed the graphene oxide layers intercalated or embedded with ca.20 nm TiO_2_ particles in the TGO-20% sample, in which the graphene oxide consisted of quite a few layers.

These loose stacking pattern could be attributed to either the stacking structure of the graphene oxide sheets or the introduction of graphene oxide that prevented the agglomeration of TiO_2_ nanoparticles, therefore increasing the surface area. The BET surface areas of TiO_2_, TGO-10% and TGO-20% are 45.2 m^2^/g^−1^, 79.2 m^2^/g^−1^ and 83.6 m^2^/g^−1^ respectively, respectively. The TiO_2_/GO composites exhibit obviously larger specific surface areas than pure TiO_2_, this may due to the presence of GO in the composites, which has an extremely high surface area. The high-resolution TEM image of the composite in Fig. 1(e) showed a well-defined crystal lattice spacing of 0.354 nm, corresponding to the (101) plane of anatase TiO_2_. And as observed in the selected area electron diffraction (SAED) pattern of TGO-20% in Fig. 1(f), the clearly diffraction rings from inside to the outside were corresponding to the (101), (004), (200), (211) and (204) plane of anatase TiO_2_ separately.

**Figure 2:**
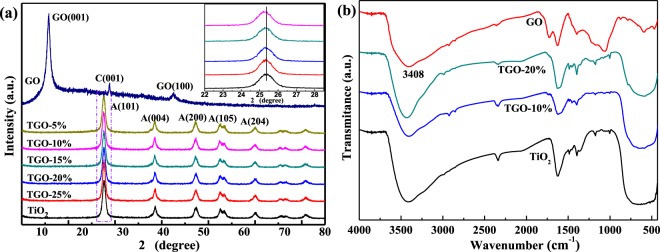
(**a**) XRD spectra of samples; (**b**) FT-IR spectra of samples.

Figure 2(a) shows the XRD patterns of GO and different TiO_2_/GO composites. The sharp peak at 11.7° is the characteristic diffraction peak of GO, corresponds to the layer spacing of GO 0.72 nm, which is much larger than that of graphite^23^. The spacing increase is caused by the introduction of a large number of oxygen-containing functional groups into the graphite structure, which results in the significant expansion of the lamellae of the graphite layer. The diffraction peaks of TiO_2_ and different TiO_2_/GO composites fit well with the anatase TiO_2_ (JCPDS: 21–1272), peaks at 25.4°, 37.9° and 48.2° corresponds to the characteristic peak of crystal plane (101), (004) and (200) of anatase TiO_2_ respectively. The presence of GO retains the anatase phase beneficially, and almost have no effect on the XRD pattern and crystallinity of TiO_2_. No diffraction peaks from GO are identified in the TiO_2_/GO composites because of the relatively small mass percentage or due to a decreased layer-attacking regularity of GO nanosheets in the composites. Moreover, the enlarged spectra (inset) clearly shows the diffraction peaks at about 25.4° is gradually moving towards the lower angle, revealing the chemical interaction between TiO_2_ and GO might exist undoubtedly.

Figure 2(b) shows the FT-IR spectrum of GO and TiO_2_/GO. The broad band at about 3408 cm^−1^ corresponds to the stretching vibrations of structural OH groups. The most obvious features in the FT-IR spectra of GO are the absorption bands attributed to the C=O carbonyl stretching at 1730 cm^−1^, the skeleton vibration absorption peak of C=C appears at 1626 cm^−1^, the bending vibration absorption peak of C-H appears at 1395 cm^−1^ and the vibration absorption peak of C-O appears at 1048 cm^−1^. All of these absorption bands confirm the graphite is fully oxidized and the presence of the functional groups -COOH on the surface of GO^24^. The absorption band at 620 cm^−1^ can be attributed to the Ti-O-Ti stretching vibration, and it is obvious that the Ti-O-Ti absorption peak is weakened in TiO_2_/GO. This proves that there may exist some interaction between TiO_2_ and GO. While the absorption peak at 3408 cm^−1^ and 1730 cm^−1^ become weaker in TiO_2_/GO further confirmed the possibility of the chemical bonding reactions, which is consistent with the XRD results.

**Figure 3:**
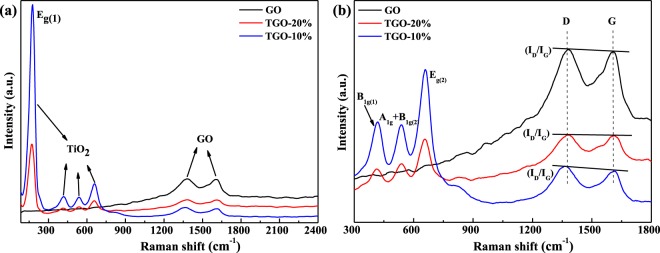
(**a**) Raman spectra of GO and GO/TiO_2_; (**b**) The partially enlarged Raman spectra of GO and GO/TiO_2_.

Raman spectroscopy is an effective tool to investigate and characterize carbonaceous materials. The Raman spectrum for GO and TiO_2_/GO (Fig. 3) shows several characterized bands. The peaks at 177 cm^−1^, 420 cm^−1^, 537 cm^−1^ and 660 cm^−1^ correspond to the Eg_(1)_, B_1_g_(1)_, A_1_g + B_1_g_(2)_ and Eg_(2)_ Raman active modes of anatase TiO_2_^25^. Raman spectra of different samples display two prominent peaks at ~1379 cm^−1^ and ~1613 cm^−1^, which correspond to the well-documented D band and G band, respectively. As shown in Fig. 3(b) the intensity ratio (I_D_/I_G_) of TGO-20% is increased, indicating the formation of more sp^3^ carbon. “The Raman peak of GO at about 1379” cm^−1^ shifted to the lower wave number with the TiO_2_ content increasing, this can be concluded that the stress induced by more TiO_2_ nanoparticles grown on surface of grapheme, which further confirms the chemical interaction between TiO_2_ and GO^26^, the surface functional groups of the TiO_2_/GO are also confirmed by FT-IR analysis and coinciding with the XRD results above.

**Figure 4:**
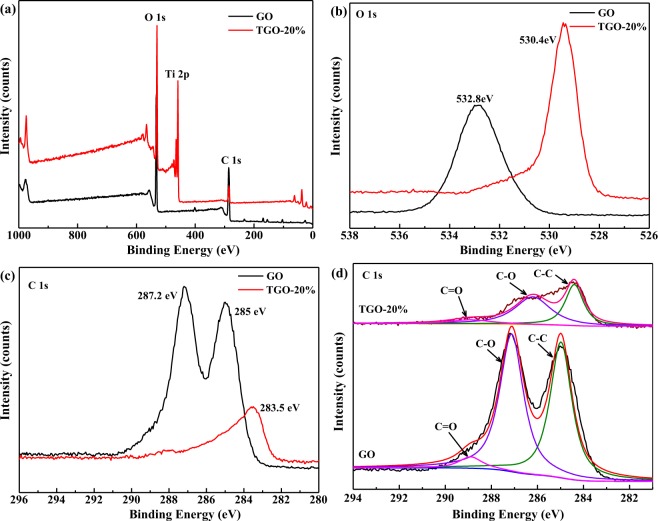
XPS spectra of GO and TGO-20% (**a**) survey scan; (**b**) O 1s; (**c**) C1s and (**d**) fitted spectra of C1s.

XPS was carried out to investigate the chemical state, element composition and distribution of the sample surface (Fig. 4). Figure 4(a) describes the full survey of spectrum of GO and TGO-20%, in which the elements of O, Ti and C can be clearly identified, Fig. 4(b–d) show the O 1 s and C1s fine spectra respectively. As shown in Fig. 4(b), the O 1s XPS spectra of GO and TGO-20% are different in peak shapes and peak positions. For GO, the peak at 532.8 eV is attributed to the hydroxyl group on the surface, and for TGO-20%, the peak at 530.4 eV can be assigned to the Ti-O-C bond. The Ti-O-C bond in TGO-20% indicates that strong interaction between GO and TiO_2_ had been formed at the hydrolysis process^27^. Figure 4(c) shows the C 1 s spectra of GO and TGO-20%, and the intensity of the C 1 s peaks of TGO-20% is much lower than that of GO. The peaks around 285 eV and 287.2 eV are attributed to the C-C and C=C bonds, while the peak centered at about 283.5 eV can be assigned to the C-O bond in GO. The deconvolution curves of the C 1 s peak of GO and TGO-20% are shown in Fig. 4(d), the deconvoluted peaks centered at 285, 287.1 and 288.9 eV are attributed to the C-C, C-O and O-C=O bonds for GO, while the peaks centered at 285, 287.1 and 288.9 eV are attributed to the C-C, C-O and O-C=O bonds^28^. As shown clearly in the spectra, the strength of oxygen-containing functional groups in TGO-20% decreased significantly, suggesting that some oxygen-containing functional groups are reduced after hydrothermal recombination of GO and TiO_2_.

**Figure 5:**
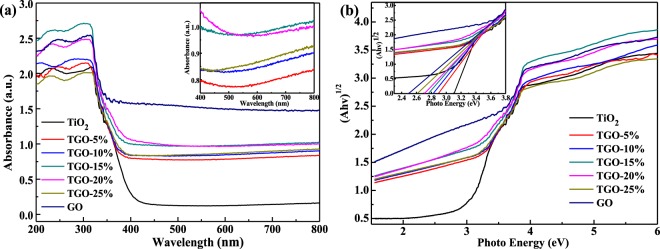
(**a**) UV-vis absorption spectra of samples; (**b**) Plots of transformed (Ahv)^1/2^ versus hv and gap energies (inset) of samples.

Figure 5 describes UV-vis absorption spectra of samples. The main absorption peak for all the samples appears at about 310 nm. GO presents a high absorption efficiency in all the ultraviolet and visible light range, TiO_2_ has low absorption efficiency in the visible light range, while all the TiO_2_/GO composites exhibit obviously enhanced absorption in the visible light region compare with TiO_2_. As for the different TiO_2_/GO composites, the visible light absorption capacity increases with the GO content increased, the absorption reaches its maximum when GO content increases to 15%, and then the absorption began to decrease as the GO content continue to increase. This result may due to the aggregation of TiO_2_ particles on the surface and more severe stacking of GO sheets into clumps. The efficient absorption in visible light implying more efficiency in exploiting the sunlight for the photocatalytic purpose, and the band gap of samples could be calculated from the formula^29^ *αhv* = *A(hv* − *Eg)*^2^. Based on the absorption spectra (Fig. 5a), plotting *(αhv)*^*1/2*^ to *hv* and then extrapolating the absorption edge onto the energy axis can give the band gap energy (Eg) of different samples, as shown in Fig. 5b and Table 1. The results obviously demonstrate the significant influence of GO on the optical characteristics of TiO_2_, the band gap of the composite decreased with the increase of GO significantly. The extended light absorption in the visible light can be ascribed to the formation of Ti-O-C chemical bonding in the prepared composites, which is further confirmed by FT-IR and XRD analysis.

**Table 1:** Kinetic date of the photodegradation process.

Sample	q_e_ (mg/g_catalyst_)	k_1_ (min·g_catalyst_/mg)	k_2_*10^−3^ (min^−1^)	R	Eg (eV)
TiO_2_	0.9675	1.18808	2.04	—	3.11
5%	8.4552	0.10649	5.27	2.58	2.91
10%	12.41	0.04787	12.75	6.25	2.84
15%	12.84	0.04053	11.22	5.50	2.76
20%	18.60	0.03458	6.75	3.31	2.71
25%	20.25	0.03393	—	—	2.62

### MB adsorption performance of TGO-x% composites

**Figure 6:**
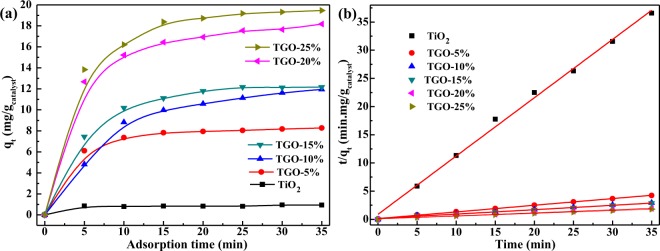
(**a**) Asorption capacity over time of different samples; (**b**) second-order kinetic plots for MB asorption.

The photocatalytic performance is significantly dependent on the adsorbability and electron transfer capacity of the photocatalyst. The study of MB adsorption by the prepared photocatalysts was performed in the absence of light radiation. The detailed adsorption information and the effect of GO are shown in Fig. 6. The amount of MB uptake of the photocatalyst q_t_ (mg/g_catalyst_) could be calculated according to the following equation: q_t_ = [(C_0_ − C_t_) × 1000 × M_W_ × V_0_]/W_catalyst_, where C_0_ and C_t_ (mol/L) are the initial concentration and concentration at time t of MB; M_W_, V_0_ and W_catalyst_ are the molecular weight (g/mol), solution volume (L) and the mass of catalyst (g), respectively^30^.

In order to study the adsorption of MB onto the surface of the prepared photocatalysts, the kinetic model: second-order model is used. The pseudo-second-order equation based on adsorption equilibrium capacity is expressed in the form^31^: ${\rm{d}}\frac{{{\rm{q}}}_{{\rm{t}}}}{{{\rm{d}}}_{{\rm{t}}}}={{\rm{k}}}_{1}{({{\rm{q}}}_{{\rm{e}}}-{{\rm{q}}}_{{\rm{t}}})}^{2}$, where q_e_ (mg/g_catalyst_) is the equilibrium adsorption capacity and k_1_ (min·g_catalyst_/mg) is the adsorption rate constant. Take t = o to t = t and q_t_ = 0 to q_t_ = q_t_ as the boundary conditions, integrating the above equation to obtain the following linear form: $\frac{t}{{q}_{t}}=\frac{1}{{{\rm{k}}}_{1}{{\rm{q}}}_{{\rm{e}}}^{2}}+\frac{1}{{q}_{e}}t$. Figure 5(b) shows the experimental data evaluated from the linear transform (t/qt) = f(t), therefore, the q_e_ and k_1_ values are determined from the slope and intercept of the fitting line and shown in Table 1. As shown in Fig. 6 and Table 1, the adsorption capacity of MB was directly proportional to the graphene oxide content, TGO-25% exhibited the best adsorptivity, and adsorption capacity reached 20.25 mg/g_catalyst_, along with the lowest k_1_ value, about 0.03393 min·g_catalyst_/mg.

The adsorbability of TGO-x% composites enhanced with the increase of graphene oxide content, while GO exhibited the highest adsorbability about MB. This may attribute to the increased oxygen-containing functional groups and the lamella, porous structure of TGO-x% composites. The higher the GO content, the better the adsorbability, the important assistance of functional groups at the edge or on the surface of graphene oxide sheets was elucidated through the better adsorbability over the TGO-20% and TGO-25% composites. Moreover, the ionic interactions between cationic dyes and the negatively charged groups can be formed in the company of abundant π-π conjugations between methylene blue molecules and the aromatic rings of graphene oxide sheets^32^, which could lead to the higher adsorptivity.

### MB total removal and photocatalytic performance of TGO-x% composites

The photocatalytic activity was investigated under visible light irradiation after dark adsorption.

**Figure 7:**
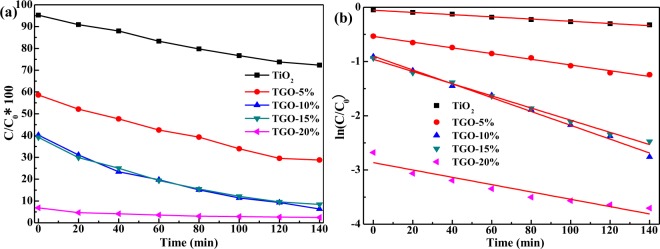
(**a**) Concentration change of MB under visible irradiation; (**b**) Linear transform ln(C/C_0_) = f(t) of the kinetic curves of MB degradation under visible illumination.

Temporal concentration change of MB solution was monitored by examining the variation in maximal absorption in the UV-vis spectra at 664 nm. The C/C_0_ of MB over different samples were presented in Fig. 7(a). When the adsorption time lasted 35 min, the adsorption rates of MB were 4.8%, 41.4%, 59.8%, 60.8% and 93.1% separately for TiO_2_, TGO-5%, TGO-10%, TGO-15% and TGO-20%. Combined with the adsorption and photocatalysis results, the C/C_0_ of MB decreased with time, and the degradation rates were 27.7%, 71.2%, 93.7%, 91.6% and 97.5% respectively for TiO_2_, TGO-5%, TGO-10%, TGO-15% and TGO-20% when the irradiation time lasts 140 min. The photocatalytic oxidation ability for MB presented to be raised with the increasing of GO content, especially when GO content is over 10%, the remarkable improvements in the dye photodegradation were observed.

Furthermore, the photodegradation reaction kinetic for MB was investigated using the apparent first-order model as follows: −ln(C/C_0_) = k_2_t, where C_0_ was the initial concentration of MB, C was the concentration of MB at time t, and k_2_ was the apparent reaction rate constant^33^. The k_2_ of TiO_2_, TGO-5%, TGO-10%, TGO-15% and TGO-20% calculated from Fig. 7(b) were listed in Table 1. Figure 7(b) and Table 1 showed the fitted kinetic results and the corresponding values. A significantly acceleration in the photo-oxidation reaction was observed, and the degradation rate of MB on TGO-x% composites was much greater than that of pure TiO_2_ and gradually accelerated with increasing the graphene oxide content up to 10%. The k_2_ values of TGO-10% and TGO-15% were 12.75 × 10^−3^ min^−1^ and11.22 × 10^−3^ min^−1^ respectively. The consequence of k_2_ was as follows: TGO-10% > TGO-15% > TGO-20% > TGO-5% > TiO_2_.

A synergy factor was defined (R = k _TGO-x%_/k _TiO2_) to estimated to quantify the extent of synergy effect of TGO-x% composites compared to pure TiO_2_^34^. The apparent reaction rate constant was been chosen as the basic kinetic parameter to compare the different systems, since it was independent on the concentration and, therefore, enabled one to determine the photocatalytic activity independently of the previous adsorption period in the dark^35^, so k_2_ was chosen to calculate R and the resulting values were listed in Table 1. As shown in the Table, the enhancement in photoactivity with synergy factor ranging from 2.58 to 6.25. The results remarkably demonstrated the kinetic synergistic effect of graphene oxide in MB photodegradation.

**Figure 8:**
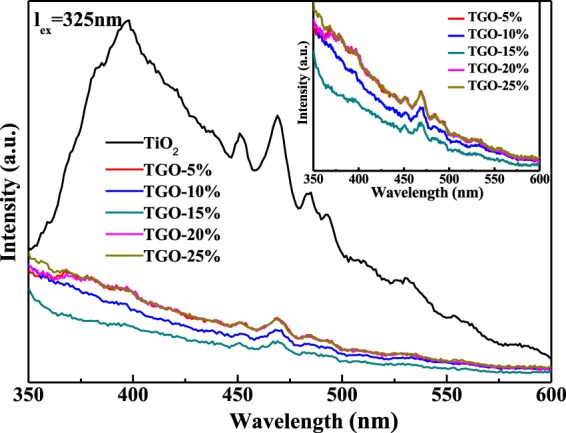
PL spectra and enlarge spectra (inset) of different samples.

PL is an effective method used to reflect the behavior of photo-induced electron-hole pairs during photocatalytic process^36,37^, the intensity of the peak mainly depends on the recombination rate of photogenerated electron and holes^38^, the recombination rate is higher, the luminous intensity is stronger. Figure 8 presented the PL spectra (λexc = 325 nm) of different samples, the peak at 450–500 nm could be attributed to the transition of charge carrier. TiO_2_ exhibited a strong emission, while the PL emission intensity of TGO-x% composites decreased markedly, which suggested that the carrier lifetime is longer in the composites, which may result in enhanced photocatalytic activity in TGO-x% composites. The luminous intensity of TGO-15% was the weakest, in consistent with the corresponding better but not the best photocatalytic efficiency among the samples. Therefore, the difference between the photocatalytic efficiency among the samples could be mostly but not wholly attributed to the difference of electron-hole recombination rates.

The beneficial role of GO in MB adsorption and photodegradation could be attributed to various factors. GO can provide more oxygen-containing functional groups for TiO_2_, and the more GO is, the much functional groups are, leading to the more ionic/electro interaction, and therefore, the better adsorptivity (Fig. 6) for MB. Meanwhile, the strong absorption ability of visible light and small band gap width of GO (Fig. 5a) makes the absorption edge redshifts and the Eg decreases of TGO-x% composites. Moreover, GO can act as an effect electron conductor and an electron acceptor that accelerates the interfacial electron-transfer process from TiO_2_, strongly hindering the recombination of charge carriers and thus improving the photocatalytic activity (Fig. 7). GO also plays the role to diffuse contaminant molecules to the phase boundary or the interface to undergo effective decomposition. Therefore, TGO-x% composites exhibit the synergy effect of adsorption and photocatalysis, resulting in highly efficient MB degradation.

## Conclusions

A series of TGO-x% composites with varying amounts of graphene oxide are synthesized via a hydrothermal method, and its photoactivity is evaluated by the photodegradation of MB under visible light. The TGO-x% composites have better adsorption and photodegradation effect than TiO_2_. TGO-20% exhibited the remarkable adsorptivity, and adsorption capacity is 18.6 mg/g_catalyst_, along with the k_1_ is about 0.03458 min·g_catalyst_/mg. TGO-20% exhibit the best degradation ability and the degradation rate is 97.5% after 35 min adsorption and 140 min degradation, which is 3.5 times higher than that of TiO_2_. The excellent property can be ascribed to the good electronic conductivity that accelerates the interfacial electron-transfer, the low recombination of photo-generated electron-hole pairs and narrowed band gap of GO in the composites.
